# Calreticulin and the Heart

**DOI:** 10.3390/cells11111722

**Published:** 2022-05-24

**Authors:** Jody Groenendyk, Wen-An Wang, Alison Robinson, Marek Michalak

**Affiliations:** Department of Biochemistry, University of Alberta, Edmonton, AB T6G 1H7, Canada; wen-an.wang@unige.ch (W.-A.W.); at24@ualberta.ca (A.R.)

**Keywords:** endoplasmic reticulum, chaperone, calreticulin, calcium

## Abstract

Calreticulin is an endoplasmic Ca^2+^ binding protein and molecular chaperone. As a cardiac embryonic gene, calreticulin is essential for heart development. The protein supports Ca^2+^-dependent signaling events that are critical to cardiomyocyte differentiation and cardiogenesis. The increased expression of calreticulin and endoplasmic reticulum/sarcoplasmic reticulum Ca^2+^ capacity produces cardiomyocytes with enhanced efficiency, and detrimental mechanical stretching of cardiac fibroblasts, leading to cardiac pathology. Deletion of the calreticulin gene in adult cardiomyocytes results in left ventricle dilation, an impaired electrocardiogram, and heart failure. These observations indicate that a well-adjusted endoplasmic reticulum and calreticulin-dependent Ca^2+^ pool in cardiomyocytes are critical for the maintenance of proper cardiac function.

## 1. Introduction

Cardiovascular disease is one of the major health burdens in developed countries. Key causes of heart failure are ischemic heart disease and myocardial infarction, which damage the heart muscle, thereby compromising heart function. Ca^2+^ is essential for normal heart function, and Ca^2+^ dysregulation is one of the hallmarks of a failing heart. Cycling of Ca^2+^ in cardiomyocytes drives muscle excitation–contraction (E-C) coupling. The sarcoplasmic reticulum (SR), a functionally specialized form of the endoplasmic reticulum (ER) and a component of the cellular reticular network, is the source of Ca^2+^ for muscle contraction and relaxation [1]. This membrane possesses a complex collection of Ca^2+^ regulatory proteins that control and regulate the E-C coupling of the cardiac muscle. Calsequestrin, the ryanodine receptor/Ca^2+^ channel (RyR), junctin, junctate, sarcalumenin, and histidine-rich protein are examples of proteins that are unique to the SR and play an important role in SR Ca^2+^ handling. Ca^2+^ release from the SR via the RyR initiates muscle contraction [2]. The muscle relaxes when Ca^2+^ is decreased in the cytoplasm by the action of SR-associated Ca^2+^-ATPase (SERCA), a plasma membrane Na^+^/Ca^2+^ exchanger, and plasma membrane Ca^2+^-ATPase [3]. As important components of the cellular reticular network, cardiomyocytes also contain functional ER, which supports important cellular functions, such as lipid biosynthesis, protein synthesis, folding, and post-translational modification. Whether these typical ER-associated functions are shared between highly organized and functionally specialized SR and the perinuclear network of ER-like membranes remains to be established [4]. The potential contribution of the ER and ER homeostasis to cardiac pathophysiology also remains to be explored. Here, we focus on calreticulin, a major Ca^2+^ binding protein of the ER, and its impact on cardiac physiology/pathology, and we provide evidence that ER functions are essential components of cardiomyocyte biology.

## 2. Calreticulin and Heart Development

Calreticulin is an ER-resident Ca^2+^-binding chaperone present in a number of diverse species [5]. The protein binds Ca^2+^ in the ER lumen with high capacity and participates in the folding of newly synthesized proteins and glycoproteins. Calreticulin, together with calnexin (an integral ER membrane chaperone similar to calreticulin) and PDIA3 (also known as ERp57), constitute a so-called “calreticulin/calnexin cycle”, which is responsible for the folding and quality control of newly synthesized glycoproteins [6,7,8,9,10]. Calreticulin is highly expressed in embryonic hearts, but despite this, the expression of calreticulin is sharply downregulated in adult cardiomyocytes, which rely on Ca^2+^ to carry out mechanical functions in the heart. Calreticulin protein contains functionally specialized domains including an N-terminal globular domain and an extended arm proline-rich domain (P-domain), which are responsible for the chaperone function of the protein. In addition, the C-terminal highly acid C-domain is responsible for Ca^2+^ binding and buffering [5].

Cardiac development is a well-controlled molecular and morphogenetic event, and even small perturbations in this process can have devastating consequences in the form of congenital heart disease [11]. In mice, calreticulin deficiency is embryonic lethal at embryonic day 14.5 due to impaired development of the ventricular wall and septum [12,13]. In vitro and in vivo biochemical and cell biological studies have indicated that Ca^2+^ handling by calreticulin is responsible for the embryonic lethality in calreticulin-deficient mice [12,14]. This finding is underscored by generating a rescue mouse model system with a constitutively active expression of calcineurin in a calreticulin-deficient mouse, which allows the development of viable embryos with live birth, indicating a critical role for calreticulin in supporting Ca^2+^/calcineurin-dependent transcriptional events during cardiac development [14]. Intriguingly, calreticulin-deficient mice that have been rescued with an expression of calcineurin have died postnatally, with marked changes in their energy metabolism being observed in the absence of calreticulin [14,15].

The ultrastructure of the myofibrils is disorganized in the developing heart in the absence of calreticulin, and the transcriptional function of MEF2C shows impaired nuclear localization, further supporting the involvement of a critical Ca^2+^ and calreticulin-dependent checkpoint in cardiac myofibrillogenesis [13]. A role for calreticulin in cardiogenesis is further supported by studies with calreticulin-deficient embryonic stem (ES) cells [13]. In the absence of calreticulin, there are impaired Ca^2+^-dependent transcriptional activities and impaired myofibrillogenesis due to decreased activity of the muscle-specific transcription factor MEF2C [13]. Inhibition of Wnt signaling is necessary to maintain ES cells in a pluripotent state [16]. In calreticulin-deficient ES cells, Wnt signaling is disrupted, indicating the importance of calreticulin and Ca^2+^ signaling during early cardiac development [17]. Calreticulin-deficient ES cells remain either pluripotent and/or in an undefined state so they are unable to properly differentiate into cardiomyocytes. Furthermore, calreticulin-deficient ES cells express a specific set of miRNAs [18].

miRNA plays a significant role in cardiovascular differentiation, function, and disease and has been the target of intense scrutiny [19,20,21]. Ingenuity Pathway Analysis of miRNA that was identified in calreticulin-deficient ES cells indicates that the top canonical pathways affected in the absence of calreticulin are Wnt signaling, TGFβ signaling, and cardiac hypertrophy markers (Table 1). One of the main families of miRNA affected in the absence of calreticulin is the miR-302 family [17,22]. The miR-302 family is a polycistronic group nestled on chromosome 3 that can induce and maintain ES cell pluripotency [22]. Recent studies demonstrate that in a human mast cell line (HMC-1 cells), miR-302e decreased in abundance after an increase in cytoplasmic Ca^2+^ concentration, leading to inflammation and upregulation of a RelA protein, which is part of the NF-κB family [23]. In calreticulin-deficient ES cells, the miR-302 family is increased, potentially playing an anti-inflammatory role by influencing the NF-κB inflammatory pathway. Is the Ca^2+^ binding function of calreticulin involved, or is its role in ER-dependent stress responses, including unfolded protein response (UPR), responsible? Likely, both are involved. Many miRNAs are targets of the nuclease activity of the ER stress sensor IRE1α, driving apoptotic events that are due to ER stress [24]. In fibroblasts that are deficient in the plasma membrane Ca^2+^ channel ORAI1, which is responsible for Ca^2+^ entry from the extracellular space due to store-operated Ca^2+^ entry, there is an increase in several miRNAs with target degradation that is dependent on intracellular Ca^2+^ levels [25].

Evidence of the importance of ER Ca^2+^ and the Ca^2+^ binding function of calreticulin in cardiac development comes from studies of GRP94, another ER Ca^2+^ binding chaperone and resident protein. GRP94 and calreticulin have many similar features. Firstly, GRP94 has a highly acidic C-terminal domain that binds approximately 20 moles of Ca^2+^ per mole of protein [26]. Second, GRP94 deficiency in mice is embryonic lethal due to impaired cardiac development [27]. Lastly, GRP94-deficient ES cells are unable to differentiate efficiently into cardiomyocytes [27]. This provides additional evidence that the Ca^2+^ binding function of calreticulin and proper ER Ca^2+^ homeostasis are essential for cardiac development.

**Figure 1:**
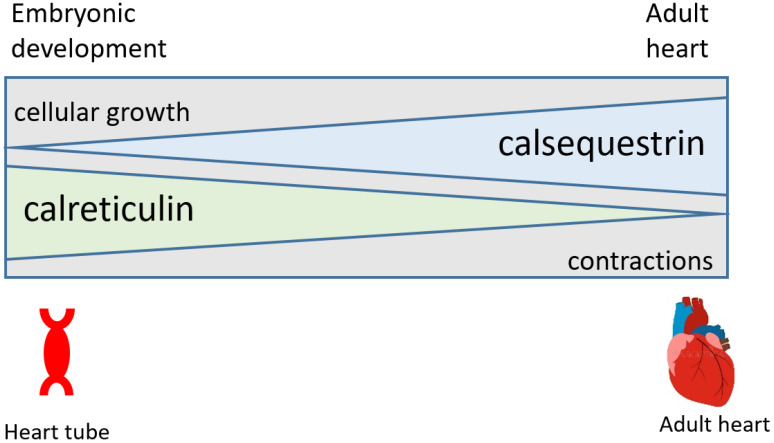
Schematic representation of a relationship between the expression of calreticulin and cardiac development. Calreticulin is abundant in the developing heart, and the expression of the calreticulin gene declines during cardiogenesis. In the adult heart, calreticulin is only a minor Ca^2+^ binding protein and calsequestrin is a major SR Ca^2+^ binding and storage protein. Calreticulin deficiency is embryonic lethal in mice. In the adult heart, either an increased abundance of calreticulin or calreticulin deficiency leads to cardiac pathology and heart failure.

Recently, somatic mutations of the calreticulin gene were discovered in patients with essential thrombocythemia and primary myelofibrosis [28]. The most common mutations were a 52-bp deletion (del52) and a 5-bp insertion (ins5), both of which led to a frameshift and a modified Ca^2+^ binding C-domain [28]. Calreticulin mutations result in the loss of the amino acid sequence (KDEL) that is responsible for ER retrieval and changes from negatively charged Ca^2+^ binding residues to a large cluster of positively charged amino acids in the C-domain [28]. Importantly, a homozygous mouse model for knock-in of the del52 mutant (no acidic residues in the Ca^2+^ binding C-domain) is embryonic lethal in a way similar to that seen for silencing of the calreticulin gene [12,29]. These findings fully support the conclusion that the loss of Ca^2+^ binding to calreticulin that affects cellular Ca^2+^ signaling is sufficient to induce embryonic lethality in mice [12,14]. This further strengthens our notion that ER-associated Ca^2+^-dependent events are critical during cardiogenesis and play different roles in fully differentiated cardiomyocytes (Figure 1).

## 3. Calreticulin in the Adult Heart

Calreticulin is highly expressed in the developing heart, but it is only a minor component in an adult heart [30]. Interestingly, an increased abundance of calreticulin in adult hearts is associated with failing and hypertrophied human hearts [31,32]. In mice, forced overexpression of calreticulin in cardiomyocytes increases cardiomyocyte ER/SR Ca^2+^ capacity and mechanical work potential but also activates the IRE1α branch of the UPR and eventually leads to cardiomyopathy [33,34]. Calreticulin overexpression also causes a reduction in the abundance of gap junction protein in the heart, indicating a defect in cell–cell communication [33]. There is an impaired expression of Ca^2+^ signaling proteins such as triadin and junctin, as well as the gap junction proteins connexin 43 and 45. Ca^2+^-handling proteins such as calsequestrin, SERCA, and the RyR, are downregulated in calreticulin-overexpressing hearts, while calmodulin, calcineurin, and MEF2C are increased [33]. Additionally, impaired gap junctions, aberrant Ca^2+^ signaling, and arrhythmia have been observed in a non-inducible calreticulin overexpression mouse model system [35]. While increasing calreticulin abundance in adult cardiomyocytes improves ER/SR Ca^2+^ capacity and delays store-operated Ca^2+^ entry, it also stimulates the UPR, which promotes an increase in cardiac TGFβ abundance that, in turn, induces increased collagen deposition and severe cardiac fibrosis [33,34]. This is due to the mechanical stretching of cardiac fibroblasts because of enhanced cardiomyocyte efficiency and the activation of the IRE1α branch of the UPR pathway [36]. Interestingly, inhibition of IRE1α activation with tauroursodeoxycholic acid (TUDCA), a proteostasis promoter [37], prevents the development of cardiac fibrosis in hearts that are overexpressing calreticulin [34,38].

Considering that calreticulin was initially identified as a component of the fetal gene program in the heart, it is not surprising that up-regulation of the calreticulin gene induces cardiac remodeling. Activation of the fetal gene program is an adaptive state that supports intrinsic cell survival pathways in the heart [39,40]. The fetal gene program is normally active during embryonic development and is necessary for the embryo to survive under hypoxic conditions. As the heart grows in utero, it is subjected to increases in hemodynamic load, a low oxygen environment, and a changing metabolic landscape. The decrease in oxygen tension specifically turns on a transcription factor, HIF1α, which is stabilized under low oxygen conditions. HIF1α targets a specific subgroup of promoters for a variety of proteins, including proteins involved in proliferation, metabolism, and angiogenesis [41]. One of these downstream targets is calreticulin [42]. Increased abundance of calreticulin likely triggers changes in the metabolic capacity that lead to a disruption in reactive oxygen species and fluctuations in Ca^2+^ uptake and efflux from the mitochondria, as well as disruptions in cellular Ca^2+^ homeostasis in general. Increased abundance of calreticulin also provides an enhanced Ca^2+^ binding/buffering capacity in the lumen of the ER, thereby supporting homeostatic recovery. Alternatively, hypoxia-induced changes in redox potential inside the lumen of the ER will affect protein translation, folding, assembly, and posttranslational modifications, triggering activation of the UPR, an ER stress-coping response, and increased expression of the calreticulin chaperone to support protein quality control events. Calreticulin expression is turned on during ER stress by Ca^2+^ signaling pathways, such as activation of the G-coupled receptors or disruption of Ca^2+^ stores. This Ca^2+^ store depletion-dependent induction of expression is reliant on new protein synthesis, implying transcriptional activation is necessary. This is likely via an ER stress element, CCAAT-N9-CCACG, that is recognized by the ER stress transcription factor ATF6 [43,44]. During ER stress, ATF6 also turns on a regulator of calcineurin called RCAN1, thereby suppressing calcineurin-dependent pathways [45]. It appears that either hypoxia or ER stress up-regulates the expression of calreticulin and fine-tunes downstream Ca^2+^-dependent transcriptional responses.

**Figure 2:**
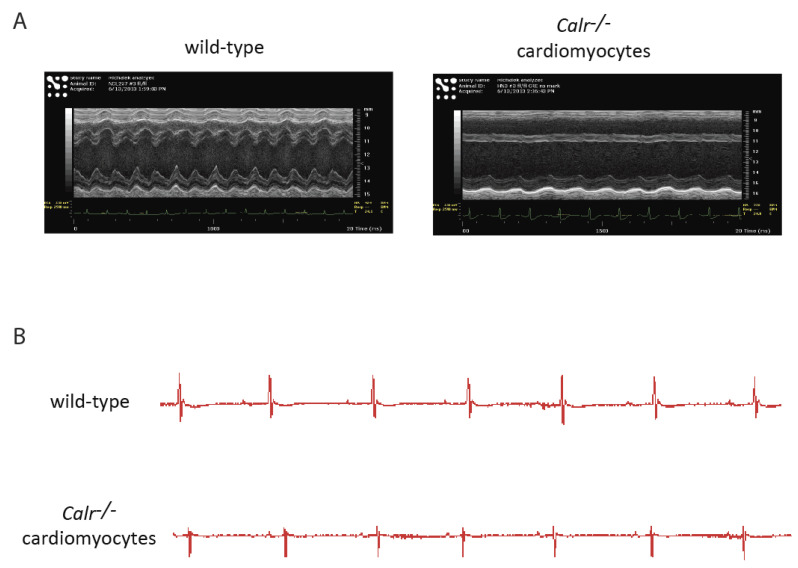
ECHO and ECG analyses of hearts with a silenced calreticulin gene in adult cardiomyocytes. Mice with a calreticulin gene containing two loxP sites flanking exons 4–7 [46] were cross-bred with αMHC (myosin heavy chain)-Cre mice (C57BL/6). To delete exons 4–7 and silence the calreticulin gene in cardiomyocytes, mice were fed tamoxifen [33]. (**A**). Representative M-mode echocardiography (ECHO) images of wild-type and calreticulin knockout (*Calr^−/−^*) hearts from mice fed tamoxifen for 2 weeks (n = 3). (**B**). Electrocardiogram (ECG) traces of electrical activity in wild-type and calreticulin knockout (*Calr^−/−^*) hearts after 2 weeks of tamoxifen treatment. Representative electrocardiography recording images of hearts from wild-type and *Calr^−/−^* mice fed tamoxifen for 2 weeks (n = 3).

**Figure 3:**
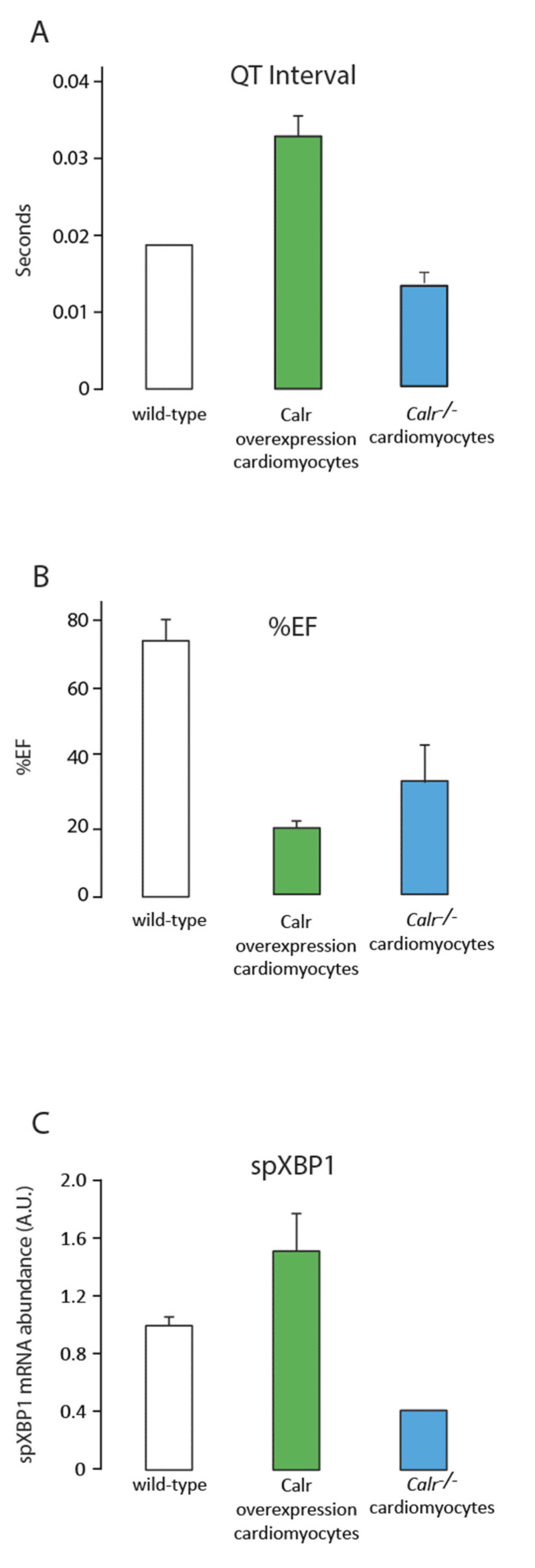
Comparison of wild-type, calreticulin-overexpressing, and *Calr^−/−^* mouse model systems. Data from three mouse models after 2 weeks of tamoxifen treatment to induce the conditional knockout of calreticulin in cardiomyocytes (*Calr^−/−^*) or 3 weeks of tamoxifen treatment for conditional overexpression of calreticulin in cardiomyocytes. (**A**). QT interval from the ECG data (Figure 2B); (**B**). percent Ejection Fraction (%EF) from the echocardiogram analysis (Figure 2A); (**C**). abundance of spliced XBP1 (spXBP1) mRNA, a measure of IRE1α activation and an ER stress sensor (n = 3).

The Cre:LoxP tamoxifen-inducible system combined with the myosin heavy-chain-promoter-driven expression of Cre recombinase [33,46,47] has been used to silence the calreticulin gene in adult cardiomyocytes. ECHO analysis of adult hearts from mice with calreticulin gene knockout showed severe left ventricle dilation (Figure 1 and Figure 2; Table 2). Interestingly, both the calreticulin-deficient mouse model and the calreticulin-overexpressing mouse model exhibited significantly reduced Ejection Fraction and Fractional Shortening (Figure 2 and Figure 3; Table 3). An electrocardiography recording (ECG analysis) revealed a reduction in the QT interval in adult hearts with a silenced calreticulin gene (Figure 1, Figure 2 and Figure 3; Table 3), while the QT interval was increased in the calreticulin-overexpressing hearts [33,48,49]. Short QT syndrome is associated with sudden death and atrial fibrillation [50]. This suggests that the calreticulin Ca^2+^ binding capacity is affecting the depolarization and repolarization of ventricle cardiomyocytes. The ECG analysis supported the idea that up-regulation of calreticulin (increased ER Ca^2+^ capacity) or deletion of calreticulin (reduced ER Ca^2+^ capacity) in the adult heart impairs systolic and diastolic functions. Calreticulin deficiency in adult cardiomyocytes has also resulted in a 50% reduction in spXBP1 mRNA compared to a 30% increase in spXBP1 when calreticulin is overexpressed (Figure 3, [34]), indicating a connection between the expression of calreticulin and activation of IRE1α, an ER stress sensor.

## 4. Conclusions and Future Challenges

1. ER Ca^2+^ capacity/homeostasis is essential for cardiac development and leads to heart disease when dysregulated.
2. Calreticulin supports Ca^2+^-dependent signaling events that are critical to cardiomyocyte differentiation and cardiogenesis.
3. Calreticulin is a major Ca^2+^ binding protein in the ER/SR of the developing heart and is downregulated after birth when calsequestrin, a muscle-specific Ca^2+^ binding/storage protein, is upregulated to supply Ca^2+^ that supports E-C coupling.
4. The increased expression of calreticulin and an increased ER/SR Ca^2+^ capacity produce cardiomyocytes with enhanced efficiency, triggering detrimental mechanical stretching of cardiac fibroblasts and leading to cardiac pathology.
5. The calreticulin-dependent Ca^2+^ pool in adult cardiomyocytes must be controlled as any increase or decrease in calreticulin results in heart pathology.
6. Resolving the specific functions of ER versus SR in muscle cells remains a challenge.
7. The role of calreticulin mutants needs to be explored for a better understanding of their role in cardiac pathophysiology.
8. Do other ER-associated chaperones play a role in cardiomyocyte Ca^2+^ homeostasis?
9. Further understanding of the role of ER/SR lumenal Ca^2+^ homeostasis will allow for the development of more targeted approaches to combat heart disease.

## Figures and Tables

**Table 1:** Ingenuity Pathway Analysis.

**Molecular and Cellular Functions**	**Number of Molecules**
Cellular Growth and Proliferation	883
Cellular Development	763
Cellular Movement	584
**Top Canonical Pathways**	**Ratio**
Wnt Signaling	40/63
TGF-β Signaling	52/86
Cardiac Hypertrophy Signaling	106/259

Ingenuity Pathway Analysis of 31 differentially expressed miRNAs in calreticulin-deficient ES cells, targeting 6942 genes. miRNA expression was analyzed in calreticulin-deficient ES cells and Ingenuity Pathway Analysis was carried out on the miRNAs that were differentially expressed.

**Table 2:** Echocardiogram Analysis.

	Wild-Type	*Calr^−/−^*	Calr OE
Body weight (g)	20.725 ± 0.245	16.285 ± 0.595	20.71 ± 0.581
% EF	75.485 ± 7.765	22.775 ± 11.875	15.40556 ± 4.430
% FS	44.035 ± 7.125	10.5 ± 5.710	10.62471 ± 2.200
LV Mass (g)	73.875 ± 0.615	66.22 ± 8.650	85.10569 ± 4.049

Calreticulin-deficient hearts (*Calr^−/−^*); hearts with an increased abundance of calreticulin (Calr OE); ejection fraction (EF); fractional shortening (FS); left ventricle (LV).

**Table 3:** Electrocardiogram Analysis.

		Wild-Type	*Calr^−/−^*	Calr OE
RR Interval	(s)	0.152 ± 0.013	0.159 ± 0.034	0.132 ± 0.011
Heart Rate	(BPM)	403.173 ± 32.553	388.470 ± 70.599	486.023 ± 50.192
PR Interval	(s)	0.036 ± 0.004	0.038 ± 0.003	0.031 ± 0.005
P Duration	(s)	0.017 ± 0.004	0.019 ± 0.001	0.013 ± 0.003
QRS Interval	(s)	0.009 ± 0.001	0.009 ± 0.002	0.013 ± 0.001
QT Interval	(s)	0.020 ± 0.002	0.017 ± 0.002	0.031 ± 0.004
JT Interval	(s)	0.010 ± 0.004	0.008 ± 0.001	0.016 ± 0.005
P Amplitude	(mV)	0.068 ± 0.033	0.085 ± 0.010	0.060 ± 0.032
ST Height	(mV)	0.063 ± 0.035	0.046 ± 0.025	−0.170 ± 0.147

Calreticulin-deficient hearts (*Calr^−/−^*); hearts with an increased abundance of calreticulin (Calr OE); RR Interval (the time elapsed between two successive R waves); Beat per minute (BPM); PR Interval (the time between atrial depolarization and ventricular depolarization); P Duration (the period that covers the earliest deflection to the latest deflection); QRS Interval (ventricular contraction); QT Interval (the beginning of ventricular depolarization to the end of ventricular repolarization); JT Interval (the period of time that covers the end of the J wave to the end of the T wave); P Amplitude (the height of the initial deflection P wave); ST Height (the height between the bottom of the S dip to the top of the T wave).

## Data Availability

Data Available on Request.
